# Periodontitis is associated with subclinical cerebral and carotid atherosclerosis in hypertensive patients: A cross-sectional study

**DOI:** 10.1007/s00784-023-04958-8

**Published:** 2023-04-01

**Authors:** María Vázquez-Reza, Iria López-Dequidt, Alberto Ouro, Ramón Iglesias-Rey, Francisco Campos, Juan Blanco, Manuel Rodríguez-Yáñez, José Castillo, Tomás Sobrino, Yago Leira

**Affiliations:** 1 Periodontology Unit, Faculty of Odontology and Medicine, University of Santiago de Compostela, Rúa Entrerríos SN, 15782 Santiago de Compostela, Spain; 2 Stroke Unit, Neurology Department, University Clinical Hospital, Santiago de Compostela, Spain; 3 NeuroAging Laboratory (NEURAL) Group, Clinical Neurosciences Research Laboratories, Health Research Institute of Santiago de Compostela, University Clinical Hospital, Santiago de Compostela, Spain; 4 Neuroimaging and Biotechnology Laboratory (NOBEL) Group, Clinical Neurosciences Research Laboratories, Health Research Institute of Santiago de Compostela, University Clinical Hospital, Santiago de Compostela, Spain; 5 Translational Stroke Laboratory (TREAT) Group, Clinical Neurosciences Research Laboratories, Health Research Institute of Santiago de Compostela, University Clinical Hospital, Santiago de Compostela, Spain

**Keywords:** Atherosclerosis, Cerebrovascular disease, Hemodynamics, Periodontitis, Ultrasonography

## Abstract

**Objective:**

To examine the relationship between periodontitis and subclinical intracranial atherosclerosis. The association of periodontitis with preclinical markers of atherosclerosis in other vascular territories was also explored.

**Material and methods:**

This was a cross-sectional study where 97 elderly subjects with a previous history of hypertension received an ultrasonographic evaluation to assess subclinical atherosclerosis in different vascular territories: (1) cerebral [pulsatility (PI) and resistance index (RI) of the middle cerebral artery], (2) carotid [intima-media thickness (IMT)], and (3) peripheral [ankle-brachial index (ABI)]. Additionally, participants underwent a full-mouth periodontal assessment together with blood sample collection to determine levels of inflammatory biomarkers (leukocytes, fibrinogen, and erythrocyte sedimentation rate), lipid fractions (total cholesterol and high- and low-density lipoprotein), and glucose.

**Results:**

Sixty-one individuals had periodontitis. Compared to subjects without periodontitis, those with periodontitis showed higher values of PI (1.24 ± 0.29 vs 1.01 ± 0.16), RI (0.70 ± 0.14 vs 0.60 ± 0.06), and IMT (0.94 ± 0.15 vs 0.79 ± 0.15) (all *p* < 0.001). No statistically significant differences were found neither for ABI or for other clinical and biochemical parameters. An independent association was found between periodontitis and increased intracranial atherosclerosis (OR_adjusted_ = 10.16; 95% CI: 3.14–32.90, *p* < 0.001) and to a lesser extent with thicker carotid IMT (OR_adjusted_ = 4.10; 95% CI: 1.61–10.48, *p* = 0.003).

**Conclusions:**

Periodontitis is associated with subclinical atherosclerosis in both intracranial and carotid arteries in elderly subjects with hypertension.

**Clinical relevance:**

The association of periodontitis with intracranial atherosclerosis implies that periodontitis patients might have greater chances to develop ischemic stroke in the future.

## Introduction

Despite the huge progress made over the past decades, atherosclerotic vascular disease (AVD) still remains the leading cause of death in the world [1]. Systemic inflammation has now been recognized as a major contributor to the development of atherosclerosis [2]. In this context, it is of paramount importance to identify conditions that may predispose to this state. One of these diseases is periodontitis, defined as a multifactorial chronic oral condition characterized by microbially associated, host-mediated inflammation resulting in the formation of deep periodontal pockets with both periodontal attachment and bone loss of the affected teeth which eventually might lead to loss of the same [3].

In the last 2 decades, a bulk of epidemiological evidence has come out showing a positive association between periodontitis and AVD [4]. The biological mechanisms underlying this relationship have been reviewed in detail in the latest perio-cardio consensus report in which it is suggested that periodontitis contributes notably to increased systemic inflammation and that oral bacteria causally linked to periodontitis that are able to promote vascular inflammation can be found in the bloodstream as well as in atherosclerotic lesions [5, 6]. Moreover, recent reviews on this topic also highlighted the potential role of bacterial endotoxins and lipids in the mechanistic link between periodontitis and AVD [7–10]. Both epidemiological and mechanistic data are supported by other investigations whereby patients diagnosed with periodontitis presented with abnormal values of well-established surrogate markers of atherosclerosis such as increased carotid intima-media thickness (IMT) [11, 12] and low ankle-brachial index (ABI) [12, 13], which are strong predictors of future cardiovascular disease and peripheral arterial disease, respectively [14]. However, whether periodontitis could affect in the same way the cerebrovascular territory is unknown.

Early changes in the intracranial artery wall can be reliably identified by transcranial Doppler ultrasonography. The pulsatility index (PI) is recognized as a measure of distal flow resistance and vascular rigidity [15]. The PI in the middle cerebral artery is a non-invasive marker of cerebral arterial stiffness and represents an indirect measure of cerebrovascular disease (mainly cerebral microangiopathy) in high-vascular-risk individuals such as those with hypertension [16]. In the same way, another ultrasonographic parameter that has been widely used is the resistance index (RI), which reflects cerebrovascular resistance and intracranial compliance [15].

Hitherto, research on the periodontitis-stroke association has focused on clinical cerebrovascular events [17], but the potential link of periodontitis with subclinical measures of cerebral atherosclerosis has not been investigated. Our a priori hypothesis was that periodontitis would be associated with increased PI and RI, thus providing evidence that periodontitis might be involved in the development of early atherosclerotic vascular changes in the cerebral arteries. Therefore, the aim of our study was for the first time to test the relationship between periodontitis and subclinical intracranial atherosclerosis in patients at high vascular risk. In addition, the association of periodontitis with subclinical signs of atherosclerosis in other vascular territories was also assessed.

## Materials and methods

### Study sample

This was a cross-sectional study carried out between 2016 and 2019 at the University Clinical Hospital of Santiago de Compostela (Spain) and performed following the STROBE (Strengthening the Reporting of Observational Studies in Epidemiology) guidelines [18]. Dentate elderly (aged ≥ 60 years) subjects of both genders with a diagnosis of primary hypertension [19] (at least 5 years of disease evolution) were included in the study. All participants were recruited from two primary care centers in A Estrada and Porto do Son (Galicia, Spain) and referred for a detailed examination to the University Clinical Hospital of Santiago de Compostela (Spain). The following exclusion criteria were as follows: (a) < 10 teeth present (periodontal examination unreliable); (b) previous history of cerebrovascular disease, cardiovascular disease, dementia, malignancy, or other severe medical condition; (c) periodontal treatment in the last year; (d) active infectious/inflammatory diseases (e.g., HIV, hepatitis, tuberculosis, rheumatoid arthritis, allergies, or asthma); (e) treatment with systemic antibiotics, corticosteroids, and/or immunosuppressive agents within 3 months prior to periodontal examination; (f) not able to consent. The present study was conducted in accordance with the World Medical Association Declaration of Helsinki (2013) and approved by the Ethics Research Committee of Santiago-Lugo (protocol #2016/399). Written informed consent was obtained from all included participants.

### Clinical examination

24-h ambulatory blood pressure monitoring (ABPM) was performed in all participants. Mean 24-h systolic blood pressure (SBP) and diastolic blood pressure (DBP) values were recorded. Body weight was measured to the nearest 1 kg, and height was recorded to the nearest centimeter. Body mass index (BMI) was calculated with the formula weight (kg)/height (m)^2^. Self-reported classical vascular risk factors (i.e., tobacco and alcohol consumption, diabetes, and hypercholesterolemia) were also recorded together with education level.

### Ultrasonographic examination: assessment of subclinical atherosclerosis

For the study of intracranial atherosclerosis, brain hemodynamics were evaluated by determining the blood flow velocity of the right middle cerebral artery using transcranial Doppler. The PI and RI of this intracranial artery were calculated according to the following formulas: PI = (Vmax − Vmin)/Vmean and RI = (Vmax − Vmin)/Vmax. The presence of PI ≥ 1.3 and/or RI ≥ 0.7 was associated with an increased risk of major adverse cerebrovascular events [20].

To evaluate the existence of carotid atherosclerosis, the carotid IMT was measured as previously described [21]. Briefly, the image was focused on the posterior (far) wall of the left carotid artery. A minimum of four measurements of the common carotid far wall were taken 10 mm proximal to the bifurcation to derive the mean carotid IMT [22]. The presence of an atheroma plaque was evaluated in the common and internal carotid extracranial arteries as well as the bifurcations according to standardized scanning and reading protocols [23]. Plaque was defined as a focal structure that encroaches into the arterial lumen by at least 0.5 mm or 50% of the surrounding IMT value or demonstrates a thickness > 1.5 mm as measured from the media-adventitia interface to the intima-lumen interface. The presence of carotid subclinical atherosclerosis was defined as an IMT value > 0.89 mm in men and > 0.82 mm in women [24].

In order to assess the potential presence of peripheral atherosclerosis, the ABI was used [25] which corresponds to the ratio of ankle SBP to brachial SBP, and for the purpose of the present study, this was calculated for right and left legs using the higher value of the right or left brachial SBP as the denominator. The presence of peripheral atherosclerosis was defined as an ABI value < 0.90.

The same explorer (IL-D), blinded to clinical data, performed the ultrasonographic study using high-resolution B-mode ultrasound (Toshiba Medical Systems Corporation, Otawara-SHI, Japan) with a 7.5 MHz, linear-array transducer (Linear array transducer PLT-704AT, Toshiba, Tochigi, Japan; Phased array transducer PST-20CT, Toshiba, Tochigi, Japan).

### Periodontal assessment

Clinical periodontal parameters recorded included pocket depth (PD), clinical attachment level (CAL), dental plaque accumulation, and gingival bleeding, as previously described [21]. The presence of periodontitis was established when ≥ 2 interproximal sites with CAL ≥ 3 mm and ≥ 2 interproximal sites with PD ≥ 4 mm (not on the same tooth) or 1 site with PD ≥ 5 mm were present [26]. Additionally, a measure of periodontitis activity, the periodontal inflamed surface area (PISA), which reflects the surface area of bleeding pocket epithelium in mm^2^, was calculated [27].

Full mouth periodontal assessments were done by a trained periodontist (YL) who was previously calibrated [17]. All measurements were performed using a calibrated University of North Carolina periodontal probe (UNC15, Hu-Friedy, Chicago, IL, USA) at six sites per tooth (excluding third molars).

### Biochemical analysis

Fasting blood samples were obtained in the morning at the same time as the periodontal assessment and interview. Briefly, 2 mL of venous blood was collected from the antecubital fossa by venepuncture using a 20-gauge needle with a 2-mL syringe. Blood samples were allowed to clot at room temperature, and after 1 h, serum was separated by centrifugation (15 min at 3000 g) and 0.5 mL of the extracted serum and plasma was immediately transferred to 1.5-mL aliquots. Each aliquot was stored at −80 °C until required for analysis. Biochemical parameters analyzed in the present study included the following: (1) inflammatory biomarkers: fibrinogen (mg/dL), erythrocyte sedimentation rate, (ESR) (mm/h) and leukocytes (×10^3^/mL); (2) lipid fractions (all expressed in mg/dL): total cholesterol, high-density lipoprotein (HDL) cholesterol, and low-density lipoprotein (LDL) cholesterol; (3) glucose (mg/dL).

Determinations were performed in an independent laboratory blinded to clinical data (Central Laboratory of the Clinical University Hospital of Santiago de Compostela). Clinical investigators were unaware of the laboratory results until the study had ended.

### Statistical analysis

No formal sample size calculation was performed for this study due to the lack of data on the relationship between periodontitis and intracranial atherosclerosis. Nevertheless, a post hoc power analysis based on the results obtained from our study confirmed sufficient statistical power (> 90%) to detect a mean difference of 0.23 with a standard deviation of 0.13 when comparing PI values (the study primary outcome) between periodontitis patients and participants without periodontitis.

Mean values and standard deviation (mean ± SD) were calculated for continuous variables and compared using an independent *t*-test after normality was confirmed by the Kolmogorov–Smirnov test. Non-normally distributed continuous variables were expressed as medians [P_25_, P_75_] and compared with the Mann–Whitney *U* test. Categorical data were reported as percentages (%) and compared by the chi-square test. Parametric correlation analyses between clinical periodontal parameters and ultrasonographic markers of subclinical atherosclerosis were performed using Pearson’s correlation coefficient. General linear models for the analysis of covariance were created to compare the mean values of significant ultrasonographic markers between periodontitis patients and non-periodontitis subjects adjusted for potential confounders (covariates). Logistic and linear regression models were performed to test potential associations between periodontitis and ultrasonographic parameters. All tests were performed at a significance level of *α* = 0.05. All data analyses were performed with statistical software (IBM SPSS Statistics version 24.0 for Windows, IBM Corporation, Armonk, NY, USA).

## Results

### General characteristics, periodontal and biochemical data

Ninety-seven elderly subjects with primary hypertension were included in the present study, of which 62.9% had a diagnosis of periodontitis. The characteristics of the study population are summarized in Table 1. No significant differences between periodontitis and non-periodontitis participants were observed in relation to socio-demographic and clinical variables. Also, no differences were noted for anti-hypertensive medication. All participants were under anti-hypertensive treatment for at least 5 years. The percentage of individuals showing well-controlled BP was similar in the periodontitis and non-periodontitis groups (47.5% vs 41.7%, *p* = 0.0831). Subjects in the periodontitis group were more often current smokers than those without periodontitis (26.2 vs 8.3%, *p* = 0.032). No major differences were noted for other classical vascular risk factors. Regarding biochemical parameters, levels of serum HDL were statistically significantly elevated in periodontitis subjects compared to those without periodontitis (62.65 vs 53.09 mg/dL, *p* = 0.014). No substantial differences were found for other lipids, inflammatory, and metabolic biomarkers reported in Table 1. As expected, higher levels of plaque accumulation and gingival inflammation were noticed in periodontitis patients than in subjects without periodontitis (Table 1). Also, statistically significant differences between study groups were observed with regards to cumulative measures of both past (CAL) and current periodontitis (PD and PISA) (Table 1).

**Table 1:** Study sample characteristics

	Periodontitis (*n*= 61)	No periodontitis (*n* = 36)	*p* - value
Socio-demographic variables			
Age	71.5 ± 5.12	69.9 ± 4.82	0.148
Males, *n* (%)	22 (36.1)	19 (52.8)	0.107
Low educational level, *n* (%)	38 (62.3)	21 (58.3)	0.313
Clinical variables			
BMI (kg/m^2^)	29.98 ± 4.50	31.25 ± 5.12	0.209
SBP (mmHg)	122.51 ± 11.32	124.22 ± 12.21	0.566
DBP (mmHg)	69.57 ± 7.66	72.78 ± 9.15	0.128
Anti-hypertensive treatment			
ACE inhibitors, *n* (%)	15 (24.6)	7 (19.4)	0.559
ARBs, *n* (%)	39 (63.9)	25 (69.4)	0.616
Calcium channel blockers, *n* (%)	28 (45.9)	12 (33.3)	0.224
Beta-blockers, *n* (%)	4 (6.5)	7 (19.4)	0.059
Diuretics, *n* (%)	31 (50.8)	13 (36.1)	0.307
Vascular risk factors			
Smoking habit, *n* (%)	16 (26.2)	3 (8.3)	0.032
Alcohol consumption, *n* (%)	4 (6.6)	5 (13.9)	0.229
Diabetes, *n* (%)	21 (34.4)	15 (41.7)	0.476
Dyslipidemia, *n* (%)	45 (73.8)	26 (72.2)	0.868
Periodontal parameters			
FMPS (%)	57.61 ± 19.84	39.47 ± 16.35	< 0.001
FMBS (%)	51.77 ± 19.84	27.14 ± 14.24	< 0.001
PD (mm)	3.60 ± 0.92	2.73 ± 0.71	< 0.001
Sites with PD ≥ 6 mm (%)	5.00 [1.00,22.50]	0.00 [0.00,0.00]	< 0.001
CAL (mm)	3.94 ± 1.05	3.00 ± 0.88	< 0.001
Sites with CAL ≥ 5 mm (%)	8.00 [2.50, 28.00]	0.00 [0.00, 0.00]	< 0.001
PISA (mm^2^)	816.29 ± 624.05	54.91 ± 110.55	< 0.001
Number of teeth	21.41 ± 3.77	23.47 ± 3.37	0.008
Biochemical parameters			
Fibrinogen (mg/dL)	426.47 ± 88.57	434.43 ± 66.70	0.652
ESR (mm/h)	15.00 [7.50, 20.50]	11.50 [7.00, 17.50]	0.304
Leukocytes (×10^3^/mL)	7.94 ± 2.22	7.46 ± 66.70	0.281
Glucose (mg/dL)	118.61 ± 49.70	116.92 ± 34.42	0.858
Total cholesterol (mg/dL)	197.44 ± 40.60	190.08 ± 36.87	0.375
HDL (mg/dL)	62.65 ± 19.68	53.09 ± 14.43	0.014
LDL (mg/dL)	112.17 ± 36.48	112.03 ± 31.97	0.985

*BMI*, body mass index; *SBP*, systolic blood pressure; *DBP*, diastolic blood pressure; *ACE*, angiotensin-converting enzyme*; ARBs*, angiotensin II receptor blockers; *ESR*, erythrocyte sedimentation rate; *HDL*, high-density lipoprotein; *LDL*, low-density lipoprotein; *FMPS*, full-mouth plaque score; *FMBS*, full-mouth bleeding score; *CAL*, clinical attachment level; *PD*, pocket depth; *PISA*, periodontal inflamed surface area

### Ultrasonographic data

Ultrasonographic markers of subclinical intracranial atherosclerosis differed between subjects with and without periodontitis (Table 2). In particular, the differences in the multivariate model adjusted for circulating HDL concentrations and tobacco consumption were observed for middle cerebral artery PI (estimated adjusted mean difference = 0.22; 95% CI: 0.11–0.34, *p* < 0.001) and RI (estimated adjusted mean difference = 0.10; 95% CI: 0.05–0.16, *p* < 0.001). Similarly, increased subclinical carotid atherosclerosis was observed in periodontal patients when compared to non-periodontitis subjects (estimated adjusted mean difference of carotid IMT = 0.14 mm; 95% CI: 0.07–0.21, *p* < 0.001). Consistently, the number of subjects with detectable atherosclerotic plaques was also greater in the periodontitis group compared to those without periodontitis (33 out of 61 vs 11 out of 36, *p* = 0.024). ABI was similar in both groups (*p* = 0.827) (Table 2).

**Table 2:** Markers of subclinical atherosclerosis at different vascular territories

	Periodontitis (*n*= 61)	No periodontitis (*n* = 36)	*p*-value
PI	1.24 ± 0.29	1.01 ± 0.16	< 0.001
RI	0.70 ± 0.14	0.60 ± 0.06	< 0.001
IMT (mm)	0.94 ± 0.15	0.79 ± 0.15	< 0.001
Atherosclerotic plaque, *n* (%)	33 (54.1)	11 (30.6)	0.024
ABI	1.18 ± 0.16	1.17 ± 0.12	0.827

*PI*, pulsatility index; *RI*, resistance index; *IMT*, intima-media thickness; *ABI*, ankle-brachial index

### Correlation analysis

**Fig. 1:**
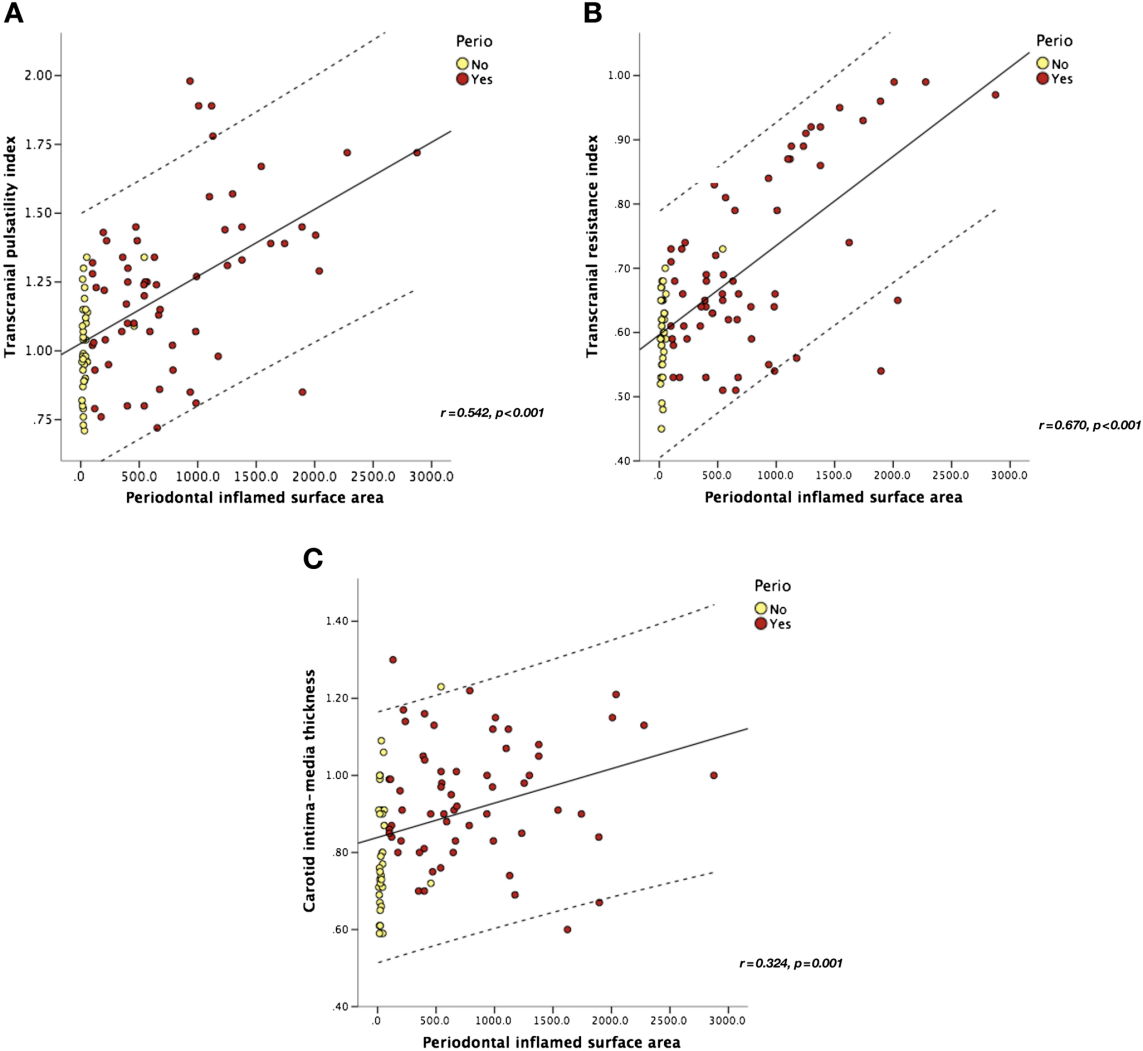
Scatter plots showing positive correlations between the periodontal inflamed surface area and **A** pulsatility index, **B** resistance index, and **C** intima-media thickness

A statistically significant positive moderate correlation was found between PISA and increased PI and RI, while the correlation was weaker with IMT (Figure 1A–C). Other clinical periodontal parameters were also correlated with PI (FMBS: *r* = 0.233, *p* = 0.022; PD: *r* = 0.377, *p* < 0.001; and CAL: *r* = 0.388, *p* < 0.001) and RI (FMBS: *r* = 0.336, *p* = 0.001; PD: *r* = 0.416, *p* < 0.001; and CAL: *r* = 0.425, *p* < 0.001) but not to IMT (FMBS: *r* = 0.160, *p* = 0.118; PD: *r* = 0.121, *p* < 0.239; and CAL: *r* = 0.119, *p* = 0.244). No correlation was found between ABI and any of the periodontal parameters (PISA: *r* = 0.105, *p* = 0.305; FMBS: *r* = 0.090, *p* = 0.381; PD: *r* = 0.082, *p* = 0.426; and CAL: *r* = 0.093, *p* = 0.363).

### Regression analysis

Results from regression analysis are shown in Table 3. Logistic regression analysis showed a positive association between periodontitis and subclinical intracranial atherosclerosis (OR_adjusted1_ = 10.16; 95% CI: 3.14–32.90, *p* < 0.001) and to a lesser extent with subclinical carotid atherosclerosis (OR_adjusted1_ = 4.10; 95% CI: 1.61–10.48, *p* = 0.003), independent of tobacco consumption and HDL serum concentrations. These results were confirmed when continuous outcomes of subclinical atherosclerosis were used in linear regression models (for mean PI: *β*coefficient_adjusted1_ = 0.22; 95% CI: 0.11–0.34, *p* = 0.003; for mean RI: *β*coefficient_adjusted1_ = 0.10; 95% CI: 0.05–0.16, *p* < 0.001; for mean IMT: *β*coefficient_adjusted1_ = 0.14; 95% CI: 0.07–0.21, *p* < 0.001). The association of the periodontitis with subclinical intracranial atherosclerosis remained statistically significant even after additional adjustment for IMT and atherosclerotic plaque (OR_adjusted2_ = 7.95; 95% CI: 2.39–26.3, *p* = 0.001; for mean PI: *β*coefficient_adjusted2_ = 0.18; 95% CI: 0.06–0.31, *p* = 0.003; for mean RI: *β*coefficient_adjusted2_ = 0.09; 95% CI: 0.03–1.53, *p* = 0.002). No statistically significant relationship was found between periodontitis and the presence of atherosclerotic plaques (OR_adjusted1_ = 2.49; 95% CI: 0.99–6.29, *p* = 0.052).

**Table 3:** Regression analysis for the association between periodontitis and markers of subclinical atherosclerosis

		OR (95% CI), *p*-value			*β*coefficient (95% CI), *p*-value		
Exposure: periodontitis (categorical)		Outcome: intracranial atherosclerosis (categorical)	Outcome: carotid atherosclerosis (categorical)	Outcome: atherosclerotic plaque (categorical)	Outcome: mean PI	Outcome: mean RI	Outcome: mean IMT
	Model 1	10.16 (3.14–32.90), *p* < 0.001	4.10 (1.61–10.48), *p* = 0.003	2.49 (0.99–6.29), *p* = 0.052	0.22 (0.11–0.34), *p* = 0.003	0.10 (0.05–0.16), *p* < 0.001	0.14 (0.07–0.21), *p* < 0.001
	Model 2	7.95 (2.39–26.3), *p* = 0.001			0.18 (0.06–0.31), *p* = 0.003	0.09 (0.03–1.53), *p* = 0.002	

*PI*, pulsatility index; *RI*, resistance index; *IMT*, intima-media thickness

Model 1: adjusted for tobacco consumption and HDL levels

Model 2: adjusted for tobacco consumption, HDL levels, IMT, and presence of atherosclerotic plaque

## Discussion

Findings from the present study suggest that periodontitis may contribute to subclinical atherosclerosis of both intracranial and carotid arteries.

The relationship between periodontitis and AVD has been extensively described in the literature. A series of meta-analyses of epidemiological studies have shown that periodontitis patients are more likely to develop coronary heart disease, ischemic stroke, and peripheral arterial disease than those without periodontitis [17, 28, 29]. Over the years, non-invasive markers of atherosclerosis have emerged [14]. The most widely used ultrasonographic method to assess subclinical atherosclerosis is the carotid IMT. Abnormal values of the IMT measured in the carotid artery are directly associated with vascular disease affecting the cerebral, coronary, and peripheral artery vascular beds [30]. Moreover, increased carotid IMT has been linked to a high risk of myocardial infarction and stroke in older subjects without a previous history of cardiovascular disease [31]. Seminal papers have demonstrated a positive relationship between periodontitis and periodontal bacteria burden with abnormal carotid IMT [11, 32, 33]. In our study, we have confirmed that periodontitis is associated with thick carotid arterial walls. However, whether periodontal treatment could reduce IMT is still unknown and deserves further investigation. On the other hand, ABI is considered a non-invasive measure to assess the patency of lower extremity arteries and establish the presence of peripheral arterial disease [14]. Also, it has been shown that a reduced ABI is an independent predictor of cardiovascular events [34]. The relationship between periodontitis and peripheral arterial disease is controversial and has inconsistent results [12, 13, 35]. In the present investigation, we did not find an association between periodontitis and reduced ABI, although a tendency was observed towards increased odds of lower ABI in patients with periodontitis.

At the cerebrovascular level, both PI and RI in the middle cerebral artery are recognized as measures of distal flow resistance and vascular wall rigidity, thus increased PI or RI may be a representative of cerebral arterial stiffness. There is a growing body of evidence suggesting that intracranial atherosclerosis and impaired brain hemodynamics are important contributors to cerebrovascular disease mainly affecting small vessels in the brain. In this sense, previous studies have suggested a relationship between increased PI and leukoaraiosis and lacunar infarction [16, 36–38]. In acute stroke patients, it has been shown that elevated PI is associated with intracranial arterial calcification [39] and that both PI and RI may be useful to discriminate ischemic stroke cases of lacunar origin [40]. As it was mentioned before, it is well established that periodontitis increases the risk of having ischemic cerebrovascular disease [17, 21]. In the present study, we observed that periodontitis patients were more likely to have intracranial atherosclerosis compared to those without periodontitis. This finding was confirmed even after adjusting for the presence of increased carotid IMT and atherosclerotic plaques. Indeed, a linear relationship was found between periodontitis and the continuous outcomes of subclinical intracranial atherosclerosis, namely PI and RI. Our results strengthen the hypothesis that periodontitis may contribute to accelerated cerebral atherosclerosis development, thus being a plausible mechanism underlying the periodontitis-stroke relationship. In a 2-year follow-up study, severe periodontitis was associated with moderate and severe grade aortic arch plaque thickness (OR = 10.5 and OR = 16.9, respectively) in patients suffering from ischemic stroke/TIA [41]. Over the study follow-up, it was confirmed that severe periodontitis increases the chances of having recurrent vascular events (HR = 2.8). In addition, multivariable regression analysis showed that severe aortic arch atheroma was associated with the presence of recurrent vascular events in ischemic stroke survivors (HR = 2.3) [41]. In the same study, those subjects with severe periodontitis had more aortic arch calcified plaques (measured by transesophageal echocardiogram) than those with no/mild periodontitis [41]. This is consistent with data from Christou and co-workers, where periodontitis increased the risk estimates for developing carotid calcifications (measured by panoramic radiographs) in ischemic stroke patients [42]. Future studies are warranted to evaluate whether periodontitis contributes to intracranial atherosclerosis in stroke survivors.

Our study has several limitations to be acknowledged. First, the present analysis is based on a cross-sectional study and hence cannot indicate or support a causal relationship between periodontitis and subclinical cerebrovascular atherosclerosis. Further evidence coming from longitudinal studies is needed to confirm our results. Second, our data is preliminary and hypothesis-generating, as no formal sample size calculation has been done. However, a post hoc power analysis based on the results obtained from our study confirmed sufficient statistical power (> 90%) to detect a mean difference of 0.23 with a standard deviation of 0.13 when compared to PI values (the study primary outcome) between periodontitis patients and participants without periodontitis. Third, participants were taking medications such as anti-hypertensives that might have affected both periodontal and ultrasonographic examinations. Perhaps future studies should include otherwise healthy participants in order to rule out the potential masked effects of medications. Finally, although PI and RI are used as markers of subclinical atherosclerosis, other ultrasonographic methods to evaluate brain hemodynamics such as cerebral vasoreactivity to hypercapnia [by means of voluntary apnea (breath-holding test) or CO_2_ inhalation technique] might be considered in the next investigations on this topic. Also, only the PI and RI from the middle cerebral artery were assessed and included in the analysis. We have only used this artery because it is the one responsible for a large proportion of the brain’s supply (2/3). Furthermore, this artery can be easily accessed through the temporal window, and the long middle cerebral artery track is suitable for evaluating peripheral resistance in the distal area in which direct examination is hardly performed [43].

## Conclusion

In conclusion, our results showed that periodontitis is associated with subclinical atherosclerosis in both intracranial and carotid arteries.

## Declarations

### Disclaimer

The funders had no role in the study design, data collection and analysis, decision to publish, or preparation of the manuscript.

## References

[1] Roth GA, Mensah GA, Johnson CO (2020). Global burden of cardiovascular diseases and risk factors, 1990-2019: update from the GBD 2019 study. J Am Coll Cardiol.

[2] Wolf D, Ley K (2019). Immunity and inflammation in atherosclerosis. Circ Res.

[3] Tonetti MS, Greenwell H, Kornman KS (2018). Staging and grading of periodontitis: framework and proposal of a new classification and case definition. J Clin Periodontol.

[4] Herrera D, Molina D, Buhlin K, Klinge B (2020). Periodontal diseases and association with atherosclerotic disease. Periodontol.

[5] Sanz M, Del Castillo AM, Jepsen S (2020). Periodontitis and cardiovascular diseases: consensus report. J Clin Periodontol.

[6] Schenkein HA, Papapanou PN, Genco R Sanz M (2020) Mechanisms underlying the association between periodontitis and atherosclerotic disease. Periodontol 2000 83:90–106. 10.1111/prd.12304 10.1111/prd.12304 32385879

[7] Pussinen PJ, Kopra E, Pietiäinen M, Lehto M, Zaric S, Paju S, Salminen A (2022). Periodontitis and cardiometabolic disorders: the role of lipopolysaccharide and endotoxemia. Periodontol 2000.

[8] Pirih FQ, Monajemzadeh S, Singh N, Sinacola RS, Shin JM, Chen T, Fenno JC, Kamarajan P, Rickard AH, Travan S, Paster BJ Kapila Y (2021) Association between metabolic syndrome and periodontitis: the role of lipids, inflammatory cytokines, altered host response, and the microbiome. Periodontol 2000 87:50–75. 10.1111/prd.12379 10.1111/prd.12379 PMC8457155 34463996

[9] Kapila YL (2021) Oral health’s inextricable connection to systemic health: special populations bring to bear multimodal relationships and factors connecting periodontal disease to systemic diseases and conditions. Periodontol 2000 87:11–16. 10.1111/prd.12398 10.1111/prd.12398 PMC8457130 34463994

[10] Meurman JH, Bascones-Martinez A (2021). Oral infections and systemic health - more than just links to cardiovascular diseases. Oral Health Prev Dent.

[11] Beck JD, Elter JR, Heiss G, Couper D, Mauriello SM, Offenbacher S (2001). Relationship of periodontal disease to carotid artery intima-media thickness: the atherosclerosis risk in communities (ARIC) study. Arterioscler Thromb Vasc Biol.

[12] Ahn Y-B, Shin M-S, Han D-H, Sukhbaatar M (2016). Periodontitis is associated with the risk of subclinical atherosclerosis and peripheral arterial disease in Korean adults. Atherosclerosis.

[13] Lu B, Parker D, Eaton CB (2008). Relationship of periodontal attachment loss to peripheral vascular disease: an analysis of NHANES 1999-2002 data. Atherosclerosis.

[14] Feinstein SB, Voci P, Pizzuto F (2002). Noninvasive surrogate markers of atherosclerosis. Am J Cardiol.

[15] Kim J (2019). Pictorial essay: transcranial Doppler findings of the intracranial and extracranial diseases. J Neurosonol Neuroimage.

[16] Sierra C, de la Sierra A, Chamorro A, Larrousse M, Domènech M, Coca A (2004). Cerebral hemodynamics and silent white matter lessions in middle-aged essential hypertensive patients. Blood Press.

[17] Leira Y, Seoane J, Blanco M (2017). Association between periodontitis and ischemic stroke: a systematic review and meta-analysis. Eur J Epidemiol.

[18] Von Elm E, Altman DG, Egger M, Pocock SJ, Gotzsche PC, Vandenbroucke JP (2008). The strengthening the reporting of observational studies in epidemiology (STROBE) statement: guidelines for reporting observational studies. J Clin Epidemiol.

[19] Williams B, Mancia G, Spiering W (2018). 2018 ESC/ESH Guidelines for the management of arterial hypertension: the task force for the management of arterial hypertension of the European Society of Cardiology (ESC) and the European Society of Hypertension (ESH). Eur Heart J.

[20] Baran J, Kleczyński P, Niewiara L (2021). Importance of increased arterial resistance in risk prediction in patients with cardiovascular risk factors and degenerative aortic stenosis. J Clin Med.

[21] Leira Y, Rodríguez-Yáñez M, Arias S (2019). Periodontitis as a risk indicator and predictor of poor outcome for lacunar infarct. J Clin Periodontol.

[22] Raitakari OT, Juonala M, Kähönen M (2003). Cardiovascular risk factors in childhood and carotid artery intima-media thickness in adulthood: the cardiovascular risk in young Finns study. JAMA.

[23] Touboul PJ, Hennerici MG, Meairs S (2007). Mannheim carotid intima-media thickness consensus (2004–2006). An update on behalf of the Advisory Board of the 3^rd^ and 4^th^ Watching the Risk Symposium, 13^th^ and 15^th^ European Stroke Conferences, Mannheim, Germany, 2004, and Brussels, Belgium, 2006. Cerebrovasc Dis.

[24] Junyent M, Gilabert R, Núñez I (2005). Carotid ultrasound in the assessment of preclinical atherosclerosis. Distribution of intima-media thickness values and plaque frequency in a Spanish community cohort. Med Clin (Barc).

[25] Aboyans V, Criqui MH, Abraham P (2012). Measurement and interpretation of the ankle-brachial index: a scientific statement from the American Heart Association. Circulation.

[26] Eke PI, Page RC, Wei L, Thornton-Evans G, Genco RJ (2012). Update of the case definitions for population-based surveillance of periodontitis. J Periodontol.

[27] Nesse W, Abbas F, van der Ploeg I, Spijkervet FK, Dijkstra PU, Vissink A (2008). Periodontal inflamed surface area: quantifying inflammatory burden. J Clin Periodontol.

[28] Blaizot A, Vergnes J-N, Nuwwareh S, Amar J, Sixou M (2009). Periodontal diseases and cardiovascular events: meta-analysis of observational studies. Int Dent J.

[29] Yang S, Zhao LS, Cai C, Shi Q, Wen N, Xu J (2018). Association between periodontitis and peripheral artery disease: a systematic review and meta-analysis. BMC Cardiovasc Disord.

[30] Burke GL, Evans GW, Riley WA (1995). Arterial wall thickness is associated with prevalent cardiovascular disease in middle-aged adults. The atherosclerosis risk in communities (ARIC) study. Stroke.

[31] O’Leary DH, Polak JF, Kronmal RA, Manolio TA, Burke GL, Wolfson SK (1999). Carotid-artery intima and media thickness as a risk factor for myocardial infarction and stroke in older adults. Cardiovascular Health Study Collaborative Research Group. N Engl J Med.

[32] Desvarieux M, Demmer RT, Rundek T (2005). Periodontal microbiota and carotid intima-media thickness: the Oral Infections and Vascular Disease Epidemiology Study (INVEST). Circulation.

[33] Söder P-O, Söder B, Nowak J, Jogestrand T (2005). Early carotid atherosclerosis in subjects with periodontal diseases. Stroke.

[34] Pamamichael CM, Lekakis JP, Stamatelopoulos KS (2000). Ankel-brachial index as a predictor of the extent of coronary atherosclerosis and cardiovascular events in patients with coronary artery disease. Am J Cardiol.

[35] Shanker J, Setty P, Arvind P (2013). Relationship between periodontal disease, Porphyromonas gingivalis, peripheral vascular resistance markers and coronary artery disease in Asian Indians. Thromb Res.

[36] Webb AJS, Simoni M, Mazzucco S, Kuker W, Schulz U, Rothwell PM (2012). Increased cerebral arterial pulsatility in patients with leukoaraiosis: arterial stiffness enhances transmission of aortic pulsatility. Stroke.

[37] Ghorbani A, Ahmadi MJ, Shemshaki H (2015). The value of transcranial Doppler derived pulsatility index for diagnosing cerebral small-vessel disease. Adv Biomed Res.

[38] Heliopoulos I, Artemis D, Vadikolias K, Tripsianis G, Piperidou C, Tsivgoulis G (2012). Association of ultrasonographic parameters with subclinical white matter hyperintensities in hypertensives patients. Cardiovasc Psychiatry Neurol.

[39] Park K-Y, Chung P-W, Kim YB, Moon H-S, Suh B-C, Yoon WT (2013). Increased pulsatility index is associated with intracranial arterial calcification. Eur Neurol.

[40] de la Cruz-Cosme C, Dawid-Milner MS, Ojeda-Burgos G, Gallardo-Tur A, Segura T (2018). Doppler resistivity and cerebral small vessel disease: hemodynamic structural correlation and usefulness for the etiological classification of acute ischemic stroke. J Stroke Cerebrovasc Dis.

[41] Sen S, Chung M, Duda V, Giamberardino HA, Offenbacher S (2017). Periodontal disease associated with aortic arch atheroma in patients with stroke or transient ischemic attack. J Stroke Cerebrovasc Dis.

[42] Christou P, Leemann B, Schimmel M, Kiliaridis S, Müller F (2010). Carotir artery calcification in Ischemic stroke patients detected in standard dental panoramic radiographs – a preliminary study. Adv Med Sci.

[43] Kassab MY, Majid A, Farroq MU (2007). Transcranial doppler: an introduction for primary care physicians. J Am Board Fam Med.

